# GlycoIP: an integrated platform for simultaneous and site-specific *N/O-*glycosylation analysis of human semen

**DOI:** 10.3389/fchem.2025.1569561

**Published:** 2025-05-19

**Authors:** Gaoshu Yan, Fei Cai, Keliang Wu, Qingyuan Cheng, Yong Zhang, Lin Fan

**Affiliations:** ^1^ Radiation Oncology Key Laboratory of Sichuan Province, Department of Radiology, Sichuan Clinical Research Center for Cancer, Sichuan Cancer Hospital and Institute, Sichuan Cancer Center, Affiliated Cancer Hospital of University of Electronic Science and Technology of China, Chengdu, China; ^2^ Department of Nephrology and Institutes for Systems Genetics, Frontiers Science Center for Disease-Related Molecular Network, West China Hospital, Sichuan University, Chengdu, China; ^3^ Department of Andrology and Sichuan Human Sperm Bank, West China Second University Hospital, Sichuan University, Chengdu, China

**Keywords:** sperm, *N-*glycosylation, *O-*glycosylation, LC-MS/MS, glycoproteomics

## Abstract

Protein glycosylation plays a pivotal role in human semen, influencing various processes, such as spermatogenesis, maturation, sperm motility, capacitation, and fertilization. Despite its importance, the specific details regarding *N/O-*glycosylation within human semen proteins have largely remained unknown. To address this challenge, an integrated glycoproteomic platform (termed GlycoIP) was developed, enabling the simultaneous analysis of both intact *N-* and *O-*glycopeptides directly from human semen samples. Characterizing these intact glycopeptides is particularly challenging, yet it provides invaluable insights into the structure and function of both glycans and their attachment sites. In this study, our platform enabled the identification of 1,833 unique *N-*glycopeptides and 720 unique *O-*glycopeptides. This approach revealed extensive and precise site-specific *N/O-*glycosylation data, highlighting 438 potential *O-*glycosylation sites from 148 distinct *O-*glycoproteins. Notably, we conducted site-specific *N/O-*glycosylation profiling on several unique glycoproteins, including sperm equatorial segment protein 1 (SPESP1), which is located on human sperm, and plasma serine protease inhibitor (SERPINA5), which is presented in both sperm and seminal plasma. In summary, this platform provides a promising approach for comprehensive profiling of protein site-specific *N/O-*glycosylation within a single experiment. This advancement paves the way for further functional studies on glycoproteins and their roles in male infertility, enhancing our understanding of this complex field.

## Introduction

Male reproductive issues, particularly those stemming from diminished semen quality, have emerged as a pressing health concern worldwide (Skakkebaek et al., 2016). Human semen is composed of sperm (S) and seminal plasma (SP), both of which are rich in secreted glycoproteins essential for male fertility (Yang et al., 2015). Among these glycoproteins, several play critical roles in human fertility, such as the sperm surface glycoprotein CD52 (Diekman et al., 1999). These glycoproteins are instrumental in various stages of male reproductive processes, including spermatogenesis, maturation, extracellular quality control, capacitation, sperm-egg recognition, and ultimately fertilization (Luo et al., 2023). Abnormal glycosylation may result in compromised semen functionality and lead to infertility. Protein glycosylation can be classified mainly into two types: *N-*glycosylation (*N-*glycans are specifically attached to asparagine (N) residues in an N-X-S/T/C motif, X≠P, proline (P), cysteine (C)) and *O-*glycosylation (with serine (S) or threonine (T)) (Zhang et al., 2018). The presence of *O-*glycosites near *N-*glycosites suggests a potential “*O-*Follow-*N*” rule (Tian et al., 2021). While this is not the main focus of our research, this observation may provide insights into the coordinated regulation of *N/O*-glycosylation events in proteins. Understanding these processes could be significant for deciphering glycosylation patterns in human semen. Exploring human semen glycoproteins could be crucial in revealing the structures and functions of these proteins, as it also contributes to our understanding of specific diseases and helps identify potential therapeutic targets (Liu et al., 2022). For example, glycodelin is a key glycoprotein in reproduction, exists in four distinct glycoforms, each serving a specific function. This means that the glycan structures attached to the protein are crucial in determining its role in fertilization processes (Seppälä et al., 2007). Research underscores the importance of these glycoproteins, indicating that both protein expression and glycan structures are likely essential to these events (Ka et al., 2019). Furthermore, previous studies have shown that *N-*glycoproteins are vital for protecting spermatozoa from immune attack in the female genital tract, as well as for their recognition and interaction with the egg (Wang et al., 2013). However, our understanding of the specific *N/O-*glycosylation patterns within these glycoproteins in human semen is still quite limited. This is primarily due to the challenges associated with accurately characterizing intact *N/O-*glycopeptides directly from human semen samples.

In recent years, the field of glycoproteomics has experienced significant advancements because of the development of cutting-edge mass spectrometers with ultra-high resolution and sophisticated software (Zhao et al., 2023; Cao et al., 2021). The methodology of glycoproteomics consists of several essential steps: selecting and processing clinical samples, conducting LC-MS/MS analysis, performing bioinformatics analysis, and verifying the results (Li et al., 2023; Zhao et al., 2024). Great progress has been achieved at each step. For example, precise analysis software has been developed to interpret glycoproteomic data. These software tools can be categorized based on variations in their core algorithms, primarily into two types: peptide-first searching tools (Byonic, MSFraggerGlyco, etc.) and glycan-first searching tools (pGlyco series) (Lee et al., 2016; Polasky et al., 2020; Liu et al., 2017). A thorough review has been carried out on their advancements, principles, and unique characteristics (Cao, 2024). Specifically, Li et al. employed the advanced Orbitrap Fusion Lumos mass spectrometer alongside StrucGP software, revealing that sialylated *N-*glycans could be crucial for the process of semen liquefaction (Li et al., 2023). This discovery opens new avenues for understanding the biochemical foundations of fertility. Similarly, Luo et al. made significant strides by compiling an extensive database on human semen *O-*glycosylation. They presented glycoproteomics based on two complementary fragmentation methods (GlycoTCFM), highlighting the power of modern analytical techniques to uncover the intricate details of *N/O-*glycosylation patterns (Luo et al., 2023). Additionally, an optimized combination of electron-transfer/higher-energy collisional dissociation (EThcD) and the stepped collision energy/higher-energy collisional dissociation (sceHCD) method (EThcD-sceHCD) developed by our team has been demonstrated to significantly increase the accuracy and depth of intact glycopeptide identification. It also complements the common fragmentation mode sceHCD and enhances the analytical capability of intact *N/O-*glycopeptides in both simple glycoproteins and complex clinical samples (Zhang et al., 2021a; Zhang et al., 2021b; Luo et al., 2022a; Mao et al., 2022; Zeng et al., 2022; Riley et al., 2020; Mao et al., 2021). These findings suggest that the analysis of intact *N/O-*glycopeptides could be key to unraveling the mysteries of male reproductive disorders (Cheng et al., 2024).

The aim of this study was to develop a comprehensive platform for the simultaneous profiling of intact *N/O-*glycopeptides from human semen. The advanced platform offers accurate and in-depth site-specific information on *N/O-*glycosylation. Detailed insights into the *N/O-*glycosylation patterns in human semen is essential for understanding their effects on semen quality and male fertility. This knowledge could be crucial for evaluating the role of glycosylation in reproductive health and may open new avenues for research on male infertility.

## Materials and methods

### Materials and chemicals

All electrophoresis reagents were purchased from Bio-Rad (Richmond, CA). Dithiothreitol (DTT), iodoacetamide (IAA), trifluoroacetic acid (TFA), formic acid (FA), and ammonium bicarbonate (NH_4_CO_3_) were obtained from Sigma (St. Louis, United States). Acetonitrile (ACN) was acquired from Merck (Darmstadt, Germany). Sequencing grade trypsin was purchased from Enzyme and Spectrum (Beijing, China). Peptide *N-*glycosidase F (PNGase F) was purchased from Sigma (St. Louis, United States). *O-*Glycoprotease (IMPa) was purchased from New England Biolabs (Ipswich, United States). Zwitterionic hydrophilic interaction chromatography (ZIC-HILIC) beads were purchased from Fresh Bioscience (Shanghai, China). The C8 extraction disks were obtained from 3 M Empore (St. Paul, United States). The Bradford protein assay kit was purchased from Thermo Fisher Scientific (Rockford, United States). Centrifugal filters (30 kDa) were purchased from Merck Millipore (Carrigtwohill, Ireland). All other chemicals and reagents were purchased from Sigma-Aldrich (St. Louis, MO, United States) or Merck (Darmstadt, Germany).

### Human semen collection

Human semen was collected from healthy donors at West China Second University Hospital of Sichuan University, Sichuan Province, China. Written informed consent was obtained from all volunteers. The human semen examination results from 40 volunteers are shown in Supplementary Table S1. The 40 semen samples were pooled and centrifuged at 4°C (2,000×g for 20 min) to separate the sperm (bottom pellets) from the seminal plasma (upper supernatant). The S and SP were collected in 10 mL tubes. This study abided by the Declaration of Helsinki principles and was approved by the Ethics Committee of West China Second University Hospital of Sichuan University.

### Protein extraction, SDS-PAGE and digestion

The obtained S and SP were processed using our optimized approach (Luo et al., 2022b). To evaluate the extraction and quantification of proteins from S and SP, these proteins were loaded onto 12% sodium dodecyl sulfate-polyacrylamide gel electrophoresis (SDS−PAGE) gels. Electrophoresis was performed at 120 V for 1 h at 4°C. The lanes were dyed using a BluPower Fast Staining Coomassie (Zoonbio, Nanjing, China). The gel was washed with deionized water. Proteins were subjected to proteolysis using a filter-aided sample preparation (FASP) protocol. Briefly, 1 mg of protein was placed in a 30-kDa filter. Following centrifugation at 13,000 × g for 15 min at 25°C, 200 μL of 8 M urea buffer containing 20 mM DTT was added, and the reduction reaction was allowed to proceed for 4 h at 37°C. Next, 50 mM IAA was added, and the mixture was incubated in the dark for 1 h at room temperature. The urea buffer was then exchanged for 50 mM NH_4_CO_3_ via centrifugation at 13,000 × g for 15 min. Finally, trypsin (20 μg) was added to each filter tube, and the proteins were digested overnight at 37°C. Peptide mixtures (Fraction 1, F1) were obtained by FASP and centrifugation at 13,000×g for 15 min. The obtained peptide concentration was measured through a colorimetric assay that quantifies the absorbance at 480 nm. Next, we used a SpeedVac to dry the peptides thoroughly. After vacuum drying, 500 μg of peptide was resuspended in 70 μL of binding buffer (80% ACN/0.2% TFA) and mixed with ZIC-HILIC (Mao et al., 2020). Following washing with binding buffer, we secured Fraction 2 (F2). Fraction 3 (F3) was subsequently acquired by elution with 0.1% TFA. Next, we processed half of F2 with 4 U of IMPa, resulting in Fraction 4 (F4). Simultaneously, treating half of F3 with 2 U of PNGase F led to the capture of Fraction 5 (F5). Additionally, by digesting half of F5 with 1 U of IMPa, we were able to produce Fraction 6 (F6).

### EThcD-sceHCD-MS/MS and sceHCD-MS/MS analysis

All the fractions were analyzed via an Orbitrap Fusion Lumos mass spectrometer (Thermo Fisher). They were separated on a column (ReproSil-Pur C18-AQ, 1.9 μm, 100 μm inner diameter, length 25 cm; Dr Maisch) over a 78-min gradient (buffer A, 0.1% FA in water; buffer B, 0.1% FA in 80% ACN) at a flow rate of 400 nL/min (0–8 min, 5–12% B; 8–58 min, 12–22% B; 58–70 min, 22–32% B; 70–71 min, 32–90% B; and 71–78 min, 90% B) and detected in the data-dependent acquisition mode. The detailed parameters of EThcD-sceHCD-MS/MS and sceHCD-MS/MS have been described previously (Luo et al., 2023).

### Data analysis

The raw data files were searched against the human UniProt database (21,007 human protein entries) using Byonic software (version 5.3.5, Protein Metrics, Inc.). The mass tolerances for the precursors and fragment ions were set at ±6 ppm and ±20 ppm, respectively. Two missed cleavage sites were allowed for trypsin digestion. Thermo Scan headers were used for fragmentation. The fixed modification was carbamidomethyl (C), and the variable modifications included oxidation (M), acetylation (protein *N-*term) and deamidation (N). In addition, the 182 human *N-*glycan compositions were specified as *N-*glycosylation. The 6 most common human *O-*glycans (HexNAc(1), HexNAc(2), HexNAc(1)Hex(1), HexNAc(2)Hex(1), HexNAc(1)Hex(1)NeuAc(1), and HexNAc(1)Hex(1)NeuAc(2)) were specified as *O-*glycosylation. HexNAc [H+]/204.087 m/z was chosen for MS/MS filtering to speed up the search. Stricter quality control methods for intact glycopeptide identification were implemented, requiring a score of no less than 300. Five monosaccharide core structures should be contained in each *N-*glycan. The identification of *N-*glycosites requires the presence of consensus glycosylation motif (N-X-S/T/C, X≠P). Data preprocessing, statistical analysis, and GO enrichment performed via our in-house Wukong platform and R packages. Model building based on the AlphaFold-predicted structure (AF-Q6UW49-F1) of sperm equatorial segment protein 1 (SPESP1) was performed using PyMOL (version 2.6.0), GlycoWorkbench (version 2.1) and GlycoSHIELD (Damerell et al., 2015; Jumper et al., 2021; Tsai et al., 2024).

## Results and discussion

### Experimental design for simultaneous profiling of intact *N/O-*glycopeptides in human semen

**FIGURE 1:**
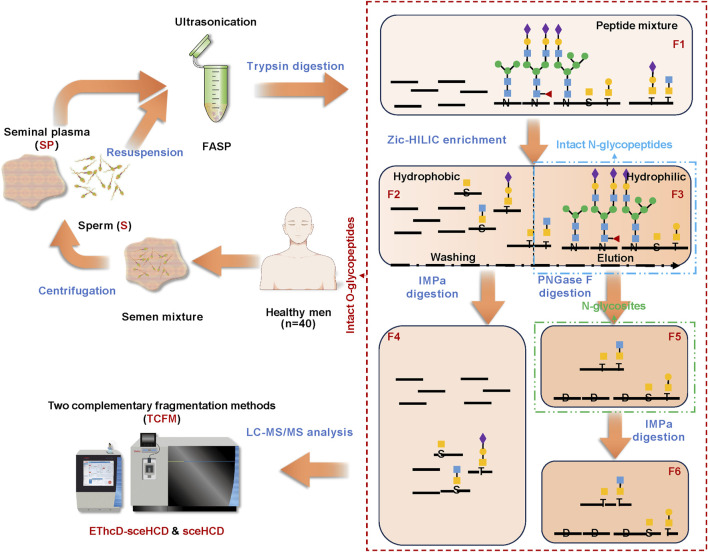
Illustration of the glycoproteomic integration platform (GlycoIP) designed for the simultaneous profiling of intact *N/O-*glycopeptides in human semen.

To achieve the simultaneous profiling of intact *N/O-*glycopeptides from human semen, we developed an innovative glycoproteomic integration platform (termed GlycoIP). As shown in Figure 1, the semen mixture from 40 healthy men (Supplementary Table S1) was separated by centrifugation into two components: sperm (S) and seminal plasma (SP). This separation is crucial for the in-depth identification and functional interpretation of *N/O-*glycosylation in human semen proteins, given the significant differences in protein composition and abundance between S and SP(32). The findings were further validated by SDS−PAGE analysis (Supplementary Figure S1). Using our optimized method for protein extraction and trypsin digestion, we obtained peptide mixture Fraction 1 (F1), which includes both high-abundance naked peptides and low-abundance *N/O-*glycosylated peptides (Luo et al., 2022b). ZIC-HILIC was subsequently used to enrich intact *N/O-*glycopeptides because it can enrich more intact glycopeptides compared to traditional HILIC (Mao et al., 2020). ZIC-HILIC contains a zwitterionic group, which enhances hydrophilic interactions with glycopeptides more effectively than traditional HILIC. This process yields two components with distinct hydrophilicities (Wang et al., 2023). F2 comprises hydrophobic peptides along with some *O-*glycopeptides, while F3 contains hydrophilic peptides and the majority of *N/O-*glycopeptides, which can be eluted sequentially from the ZIC-HILIC gradient. In comparison to *N-*glycosylation, *O-*glycosylation is less abundant and poses greater challenges for accurate identification (Zhang et al., 2018; Zhang et al., 2021c). This difficulty arises because *N*-glycosylation occurs co-translationally and is more widespread in proteins, whereas *O*-glycosylation is often post-translational and may be more restricted to specific proteins. To enable the accurate and sensitive analysis of intact *O-*glycopeptides, two strategies were employed. The first strategy involved the use of immunomodulating metalloprotease (IMPa) from *Pseudomonas aeruginosa* to digest intact *O-*glycopeptides that contain mucin-type *O-*glycans (F4 and F6). It enables accurate identification of *O-*glycan structures at each *O-*glycosite (Vainauskas et al., 2022; Suttapitugsakul et al., 2023; Helms et al., 2023; Noach et al., 2017; Kida et al., 2016; Riley and Bertozzi, 2022). Using this method, nearly 100 *O-*glycoproteins have been identified in mouse brains (Suttapitugsakul et al., 2023). However, IMPa might face a challenge where sialylation could interfere with its cleavage efficiency. The presence of sialic acid residues on glycans can obstruct the ability of IMPa to reach and break the glycosidic bonds effectively. The second strategy involves the use of peptide *N-*glycosidase F (PNGase F) for de-*N-*glycosylation, which helps to reduce sample complexity before *O-*glycopeptide analysis and ensures the identification of *N-*glycosites (F5) (Huang et al., 2015). Finally, six fractions can be obtained from each S or SP sample. The collected intact *N/O-*glycopeptide fractions were subjected to liquid chromatography tandem mass spectrometry (LC-MS/MS) analysis with both sceHCD and EThcD-sceHCD. Consequently, two complementary fragmentation methods (TCFM) were proposed for the in-depth and accurate identification of *O-*glycosylation in this study (Luo et al., 2023). Byonic was selected for its versatility in handling various fragmentation modes and its efficacy in analyzing *N/O*-glycosylation. Additionally, during a community evaluation of glycoproteomic informatics solutions, Byonic demonstrated exceptional performance in the analysis of intact *N/O-*glycopeptide data (Kawahara et al., 2021). In this study, the semen samples from all volunteers were pooled together and subsequently separated into two distinct components: S and SP. Each component was further divided into six fractions for analysis, with each fraction being examined using two different modes. As a result, a total of 24 data files were generated via the platform, allowing for the simultaneous profiling of intact *N/O-*glycopeptides from human semen in a single experiment.

### Intact *N-*glycopeptide analysis of human semen *N-*glycoproteins

**FIGURE 2:**
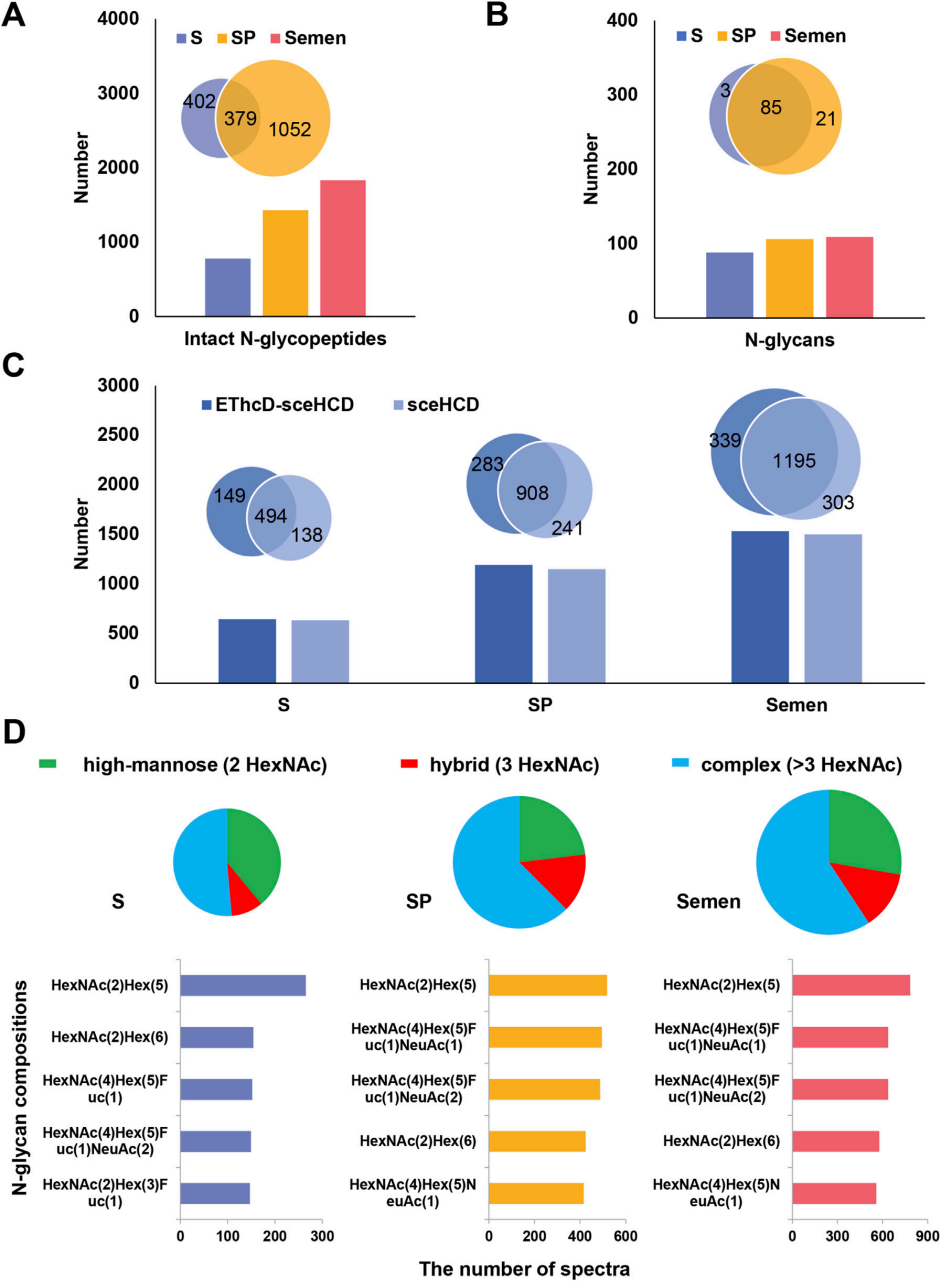
Analysis of intact *N-*glycopeptides in human semen. **(A)** Comparison of the number of identified intact *N-*glycopeptides among S, SP, and semen. **(B)** Comparison of the number of identified *N-*glycans among human S, SP, and semen. **(C)** Analysis of the number of intact *N-*glycopeptides identified by EThcD-sceHCD and sceHCD in human S, SP, and semen. **(D)** Distribution of different types (high-mannose, hybrid, and complex) and quantities of *N-*glycans in human S, SP, and semen.

Human semen contains a thick glycocalyx that is critical for sperm survival (Tecle and Gagneux, 2015). One important mechanism for regulating spermatogenesis and other functions is *N-*glycosylation (Lan et al., 2020). In this study, a total of 1,833 unique intact *N-*glycopeptides with high confidence were identified in the F3 of human semen (Supplementary Table S2). Compared with those in S (781 intact *N-*glycopeptides and 88 *N*-glycans), more intact *N-*glycopeptides (1,431) and *N-*glycans (106) were identified in SP. Furthermore, 379 intact *N-*glycopeptides and 85 *N*-glycans overlapped. (Figures 2A,B). These results suggest that human S and SP have significantly different *N-*glycosylation patterns and that these *N-*glycosylations may perform different functions, such as supporting sperm function, modulating maternal immune responses, and promoting successful fertilization (Pilch and Mann, 2006; Pang et al., 2009). Our team showed that EThcD-sceHCD complements sceHCD in terms of the number of intact *N-*glycopeptides identified from immunoglobulin G (IgG), human immunodeficiency virus type 1 envelope glycoprotein gp120 (HIV-1 gp120), hepatoblastoma cells line (HepG2), urine, plasma and tissue (Zhang et al., 2021a; Zhang et al., 2021b; Luo et al., 2022a; Mao et al., 2022; Zeng et al., 2022). In this study, EThcD-sceHCD and sceHCD were comparable and complementary in terms of the number of intact *N-*glycopeptides identified from human semen (Figure 2C). The analysis of *N-*glycans in S and SP revealed both high levels of fucosylation and sialylation. While there were some differences in the composition of the top five *N-*glycans between S and SP, HexNAc (Yang et al., 2015)Hex (Zhang et al., 2018) emerged as the most abundant *N-*glycan in both compositions (Figure 2D). Furthermore, distinct variations in *N-*glycan types were observed between S and SP. In S, the percentages of high-mannose, hybrid and complex *N-*glycan were 39.0%, 9.6%, and 51.4%, respectively. In contrast, the percentages of high-mannose, hybrid, and complex *N-*glycans in SP were 23.1%, 14.4%, and 62.5%, respectively (Figure 2D). These differences may reflect the functional differences of glycans in S and SP. Notably, glycans in SP are widely recognized for their critical role as immunomodulatory agents, as supported by the established theory of the “human fetoembryonic defense system” (Clark, 2014). These glycans contribute to immune tolerance through a mechanism that involves unique glycosylation characteristics seldom found in other bodily fluids. Specifically, SP contains specialized glycoproteins abundant in immune-related glycopeptides, which potentially serve as binding sites for endogenous lectins present on immune cell surfaces (Szczykutowicz et al., 2021).

**FIGURE 3:**
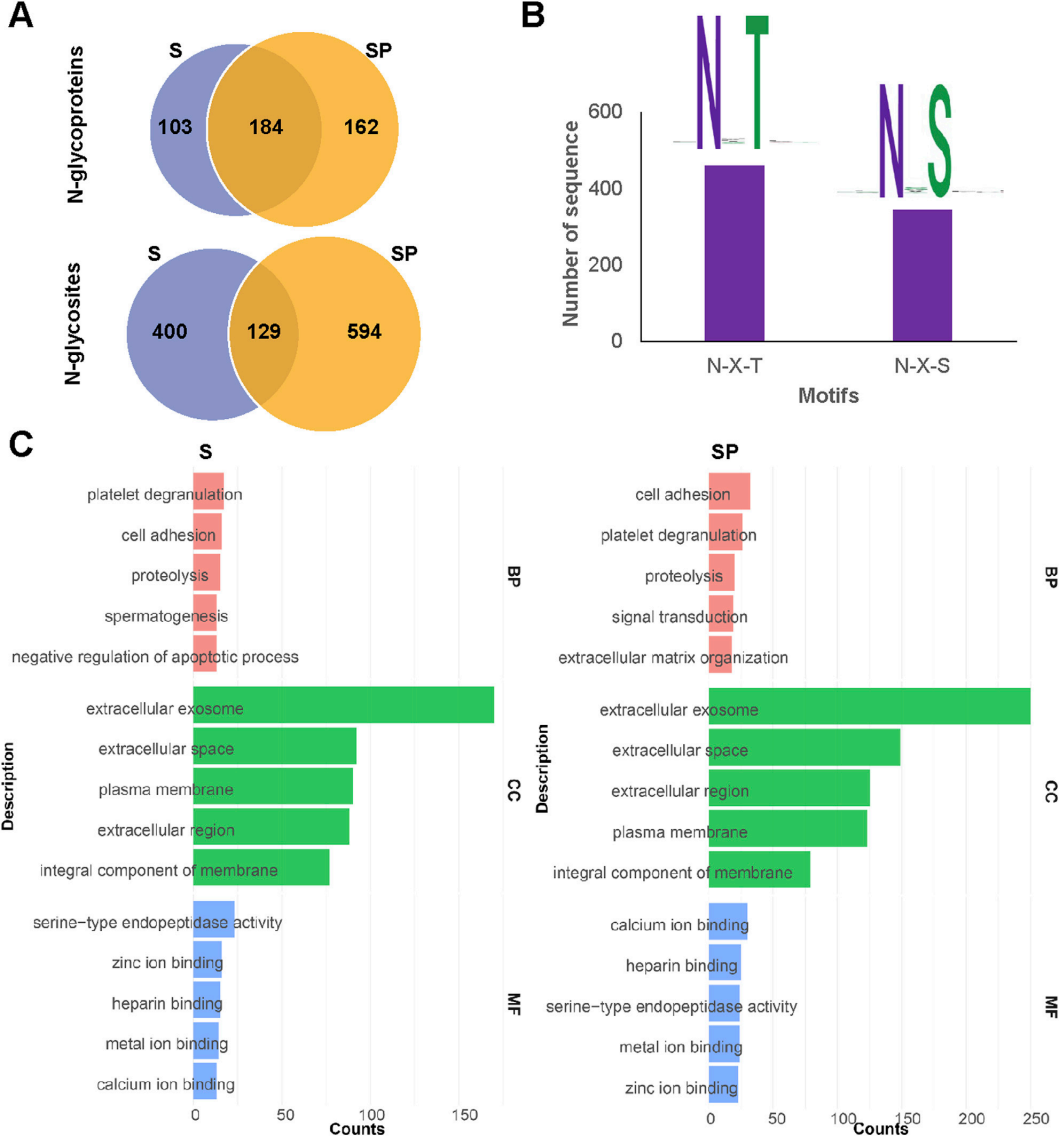
Analysis of *N-*glycosites and *N-*glycoproteins in human semen. **(A)** Comparison of identified *N-*glycoproteins and *N-*glycosites between human S and SP. **(B)** Identified *N-*glycosylated sequence motifs in human semen. **(C)** Gene Ontology (GO) analysis of biological processes (BP), cellular components (CC) and molecular functions (MF) of *N-*glycoproteins identified from S and SP.

For F5 of human S and SP, it is possible to analyze *N-*glycosites and *N-*glycoproteins. A total of 1,163 *N-*glycosites from 449 *N-*glycoproteins were identified in human semen (Supplementary Table S3). Notably, SP contained a significantly higher number of *N-*glycoproteins (346) and *N-*glycosites (723) compared to S (Figure 3A). The conserved motif N-X-S/T (X≠P) was significantly enriched, with the N-X-T motif occurring approximately 1.3 times more frequently than the N-X-S motif in both S and SP (Figure 3B). Beyond identifying these glycosites, we can also assess the occupancy of each glycosite by comparing the deamidated peptide versions to their non-amidated counterparts within the observed glycopeptides within these fractions. This comparison provides valuable insights into site occupancy, as well as micro- and macroheterogeneity. Gene Ontology (GO) analysis revealed differences in biological processes (BP) between S and SP *N-*glycoproteins, including spermatogenesis and proteolysis (Figure 3C). In recent years, the *N-*glycoproteomes of human S and SP have been investigated by LC−MS/MS (Zhang et al., 2022). For example, the GO annotations of 372 *N-*glycoproteins in human SP were located primarily in the extracellular region and associated with biological adhesion (Saraswat et al., 2016). Similarly, 297 *N-*glycoproteins in human S are located mainly in the membrane and extracellular regions and are associated with cell recognition and fertilization (Wang et al., 2013). Notably, both deamidation and deglycosylation can result in the same mass variation. Therefore, it is of great significance to conduct the identification of *N*-glycosites based on motif and UniProt database. This aligns with prior research on *N*-glycoproteins’ localization and function, hinting at potential variations in the development of biological synthesis pathways and the roles of *N*-glycosylation in human S and SP proteins (Pang et al., 2009; Saraswat et al., 2016).

### In-depth characterization of site-specific *O-*glycosylation of human semen

*O-*glycosylation has been widely reported in human samples. To date, only a few dozen *O-*glycoproteins, primarily modified by core 1 *O-*glycans, have been reported in human semen (Luo et al., 2023). In this study, we identified a total of 720 unique intact *O-*glycopeptides and 438 *O-*glycosites from 148 distinct *O-*glycoproteins present in human semen (Supplementary Table S4). Representative spectra of these intact *O-*glycopeptides from the 148 distinct *O-*glycoproteins were shown in Supplementary Table S5. To our knowledge, this is the largest number of intact *O-*glycopeptides, *O-*glycosites, and *O-*glycoproteins reported in *O-*glycoproteomic studies of human semen (Luo et al., 2023; Lan et al., 2020).

**FIGURE 4:**
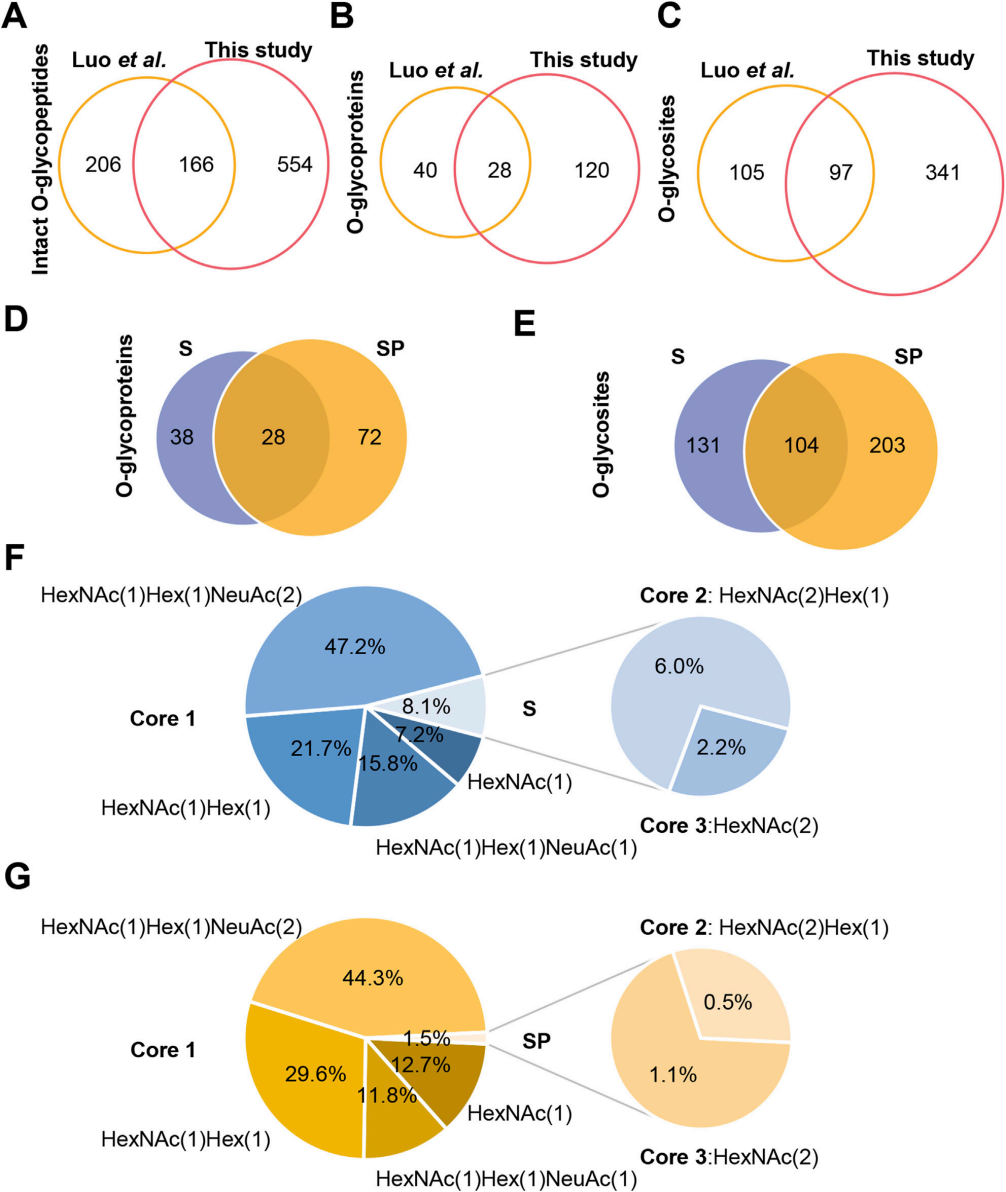
Site-specific *O-*glycosylation analysis of human S and SP *O-*glycoproteins. **(A–C)** Comparison of identified intact *O-*glycopeptides **(A)**, *O-*glycoproteins **(B)**, and *O-*glycosites **(C)** in human semen between this study and the previous study (Luo et al., 2023). **(D,E)** Comparison of identified *O-*glycoproteins **(D)** and *O-*glycosites **(E)** between human S and SP. **(F,G**) Identified *O-*glycans in human S **(F)** and SP **(G)**.

In comparison to a recent study, the depth of *O*-glycosylation identification has nearly doubled, with most of these identifications being reported for the first time (Figures 4A–C) (Luo et al., 2023). The discrepancy between the two research findings may stem from differences in sample preparation methods and analytical techniques employed for identification. Notably, the distinction in *O-*glycosylation between S and SP is significant, with 104 *O-*glycosites identified from 28 *O-*glycoproteins (Figures 4D,E). GO analysis revealed that these *O-*glycoproteins are involved in distinct biological processes. Specifically, the *O-*glycoproteins in S were involved in signal transduction and spermatogenesis (Supplementary Figure S2), whereas those in SP were involved in proteolysis (Supplementary Figure S3). Furthermore, the analysis of *O-*glycans revealed that core 1 type *O-*glycans are the most prevalent in both S and SP, with varying percentages (Figures 4F,G). Additionally, S had more core 2 type *O-*glycans (HexNAc (Yang et al., 2015)Hex (Skakkebaek et al., 2016)) and fewer Tn antigens (HexNAc (Skakkebaek et al., 2016)) than did SP (Figures 4F,G). By integrating the site-specific *N*/*O-*glycosylation information, we can gain a comprehensive and systematic understanding of protein glycosylation. However, the currently identified *O-*glycosylation may be biased due to the limitations of existing sample processing techniques (such as trypsin and IMPa), enrichment techniques (like ZIC-HILIC), mass spectrometry analysis methods (including EThcD-sceHCD and sceHCD), and software algorithms (such as Byonic) (Ahmad Izaham et al., 2021). To further improve the characterization of site-specific *O*-glycosylation in human semen, future studies could focus on developing new *O*-glycosidases capable of cleaving *O*-glycopeptides that contain sialic acids for sample processing. Additionally, utilizing more comprehensive *O*-glycan databases or open search strategies could aid in the discovery of new *O*-glycans. Furthermore, the development of more sensitive analytical techniques and software, along with conducting additional validation experiments, will be crucial.

### Distinctive glycoproteins in human sperm

**FIGURE 5:**
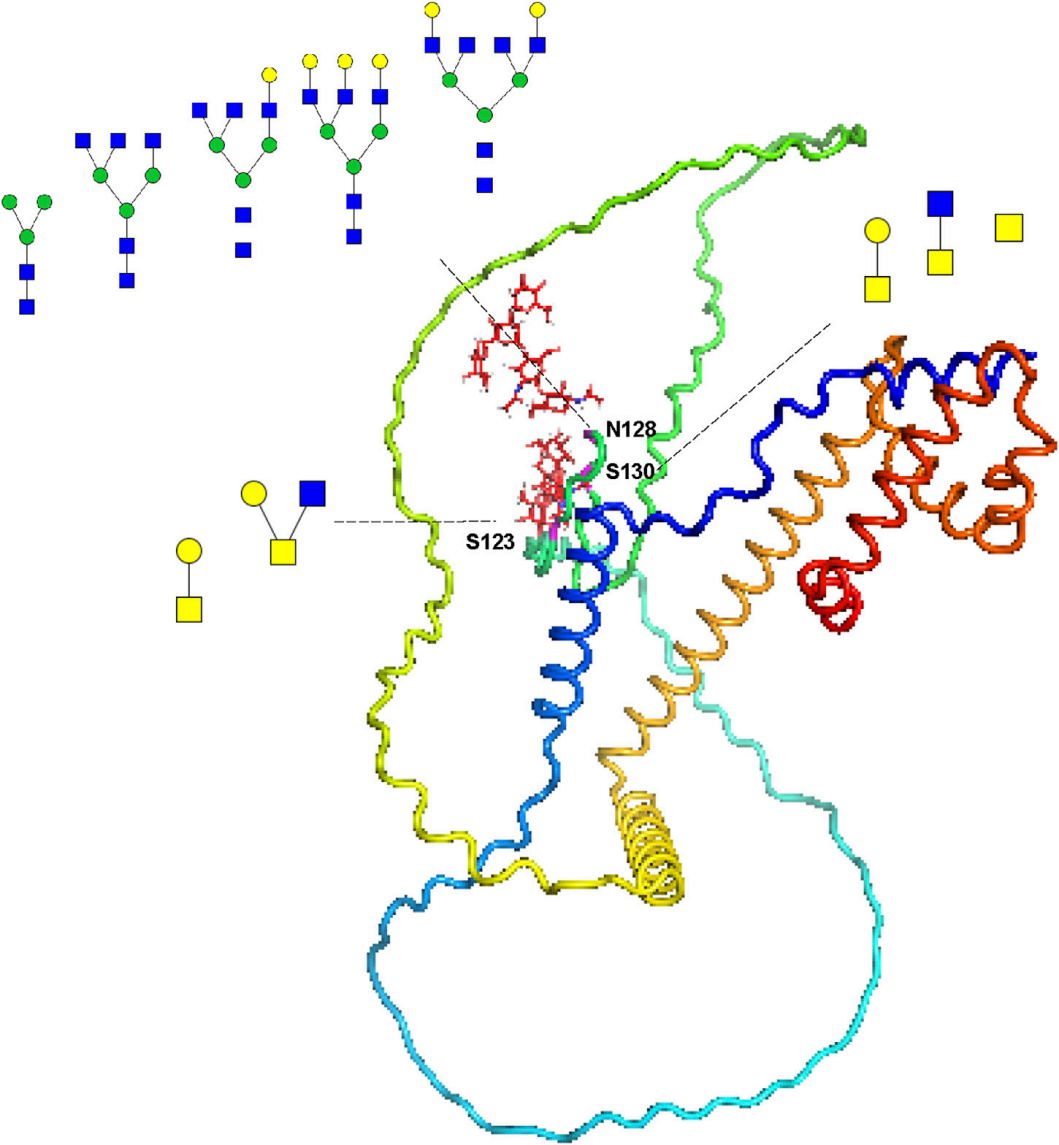
Site-specific *N/O-*glycosylation analysis of the distinctive protein sperm equatorial segment protein 1 (SPESP1) from human sperm. The AlphaFold-predicted structure of human SPESP1 was generated using PyMOL. Three glycosites (S123, N128 and S130) were identified and decorated with glycan cartoons, depicting the deduced glycan structures on the basis of their compositions.

To vividly represent site-specific *N/O-*glycosylation within a protein structure, several tools have been developed. For example, model building was conducted using the AlphaFold-predicted structures (AF-Q6UW49-F1 and AF-P05154-F1) of human sperm equatorial segment protein 1 (SPESP1) and plasma serine protease inhibitor (SERPINA5). This process utilized PyMOL (version 2.6.0), GlycoWorkbench (version 2.1), and GlycoSHIELD (Damerell et al., 2015; Jumper et al., 2021; Tsai et al., 2024). SPESP1 is a glycoprotein that is specifically and highly expressed in the testis, localized in the acrosome during the postmeiotic stages of spermiogenesis, which includes round and elongating spermatids as well as ejaculated spermatozoa. It plays an important role in the fertilization ability of sperm and serves as a suitable target for cancer immunotherapy (Wolkowicz et al., 2003; Kosaka et al., 2021). Our current analysis indicates that the protein is expressed as both an *N-*glycoprotein and an *O-*glycoprotein, primarily in S, while it was not detected in SP within the sensitivity limits of our analysis (Supplementary Table S5). Given the greater complexity and less common occurrence of glycosylation in SP, these proteins are likely present in such low quantities that they fall below our detection threshold. Our site-specific *N/O-*glycosylation analysis revealed that the *N-*glycosite (N128) can be decorated with five *N-*glycan compositions (HexNAc(2)Hex(3), HexNAc(5)Hex(3), HexNAc(5)Hex(4), HexNAc(5)Hex(6), and HexNAc(6)Hex(5)). Meanwhile, the *O-*glycosite (S123) can be decorated with two *O-*glycan compositions (HexNAc(1)Hex(1), HexNAc(2)Hex(1)), whereas the *O-*glycosite (S130) can be decorated with three *O-*glycan compositions (HexNAc(1)Hex(1),HexNAc(2), HexNAc(1)) (Figure 5).

**FIGURE 6:**
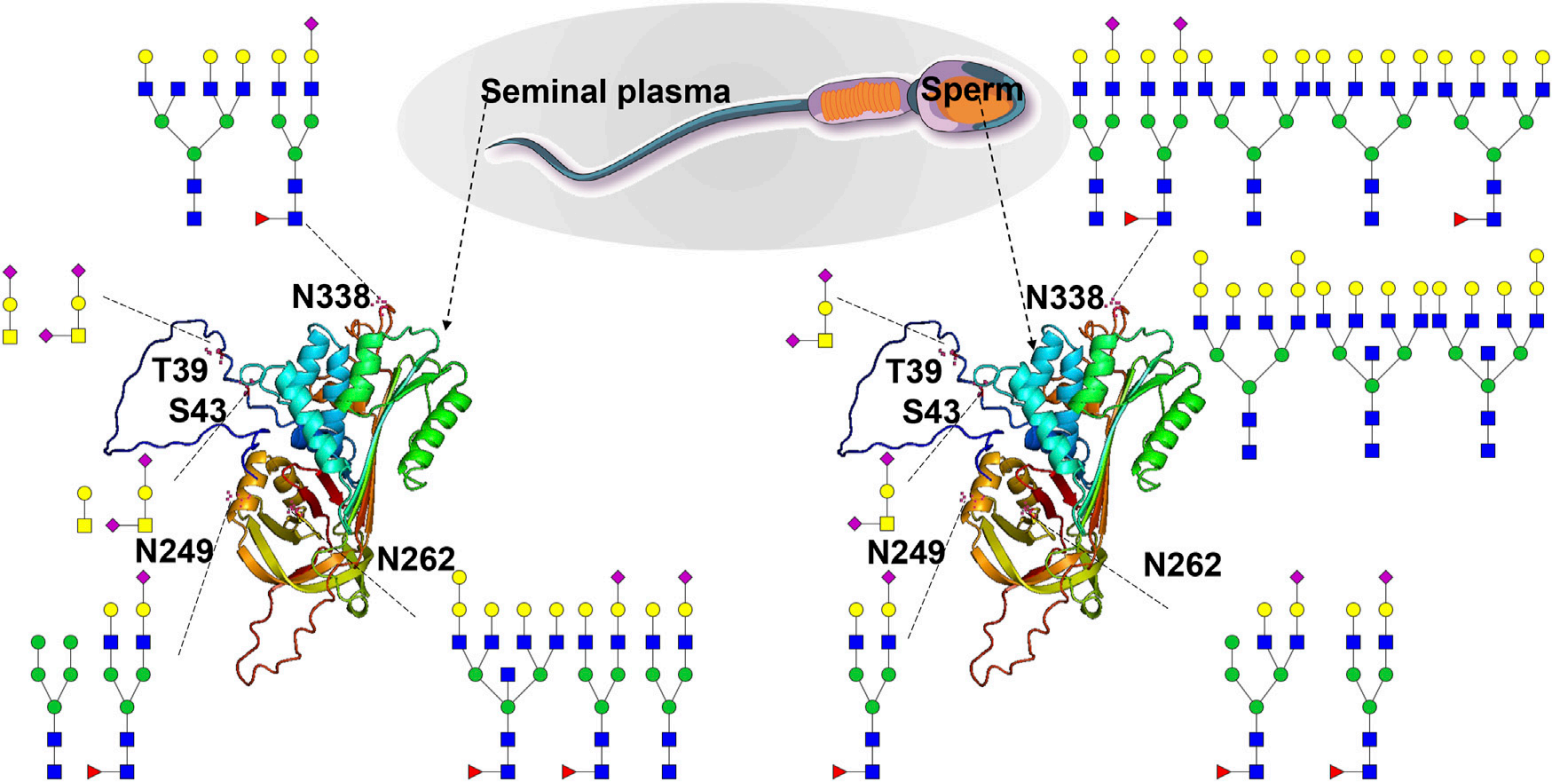
Site-specific *N/O-*glycosylation analysis of plasma serine protease inhibitor (SERPINA5) expressed in human S and SP. The AlphaFold-predicted structure of human SERPINA5 was generated using PyMOL. Three *N-*glycosites (N249, N262 and N338) and two *O-*glycosites (T39 and S43) were identified and decorated with different glycan cartoons, depicting the deduced glycan structures on the basis of their compositions.

SERPINA5 is reported to be a glycoprotein with four glycosites (T39, N249, N262, and N338) (Sun et al., 2010). It can inactivate several serine proteases involved in the reproductive system and indirectly protect components of the male genital tract from degradation by excessive release of acrosin (He et al., 1999; Jerabek et al., 2001). It also plays a role in controlling sperm motility and fertilization, as well as regulating the degradation of semenogelin during the transfer of spermatozoa from the male reproductive tract to the female tract (Cao et al., 2022; Panner Selvam et al., 2019). Our findings revealed that it is an *N/O-*glycosylated protein with fiver glycosites (T39, S43, N249, N262, and N338), and it is expressed in both human S and SP (Supplementary Table S5). Site-specific *N/O-*glycosylation analysis has shown that the reported *O-*glycosite T39 can be decorated with one *O-*glycan (HexNAc(1)Hex(1)NeuAc(2)) in sperm. In contrast, it can be decorated with two *O-*glycans (HexNAc(1)Hex(1)NeuAc(2) and HexNAc(1)Hex(1)NeuAc(1)) in seminal plasma. Notably, our GlycoIP-based research has led to the discovery of a new *O-*glycosite S43. This finding underscores the effectiveness of our method, demonstrating its ability to not only pinpoint known glycosites but also to uncover new ones. The *O-*glycosite S43 can be decorated with one *O-*glycan composition (HexNAc(1)Hex(1)NeuAc(2)), and it can be decorated with two *O-*glycan compositions (HexNAc(1)Hex(1),HexNAc(2)) (Figure 6). The detailed site-specific *N-*glycosylation information of SERPINA5 revealed that glycosylation patterns of the same protein can be differ between human S and SP. Additionally, we have included the glycan databases utilized in this study (Supplementary Table S6). These novel findings and comprehensive glycosylation data may contribute to a better understanding of glycoprotein structure and function. Additionally, our research revealed a variety of intriguing glycoproteins in S and SP, including leucine-rich repeat-containing protein 37A2, forkhead-associated domain-containing protein 1, agrin, and fibronectin. With advancements in analytical techniques, we are poised to identify an increasing number of essential seminal glycoproteins. Investigating the functions of these glycoproteins and their relationships with semen examination parameters—such as concentration, motility, morphology, genetic damage, mitochondrial function, and the acrosome reaction—will be a critical focus of future studies.

## Conclusion

In this study, we introduced an innovative platform (termed GlycoIP) designed for the comprehensive analysis of intact *N/O-*glycopeptides in human semen. The platform provides more accurate and site-specific *N/O-*glycosylation information compared to previous methods. Our findings revealed a significant number of glycopeptides and glycosites: 1,833 unique intact *N-*glycopeptides and 1,163 *N-*glycosites from 449 *N-*glycoproteins, as well as 720 unique intact *O-*glycopeptides and 438 potential *O-*glycosites from 148 *O-*glycoproteins in human semen. GlycoIP facilitates the development of a detailed, site-specific *N/O*-glycosylation map for each glycoprotein, providing valuable insights into the complex relationship between *N/O*-glycosylation patterns and male infertility. This approach not only elucidates the structural complexity of *N/O*-glycosylation but also paves the way for a better understanding of their roles in reproductive health.

## Data Availability

The datasets presented in this study can be found in online repositories. The names of the repository/repositories and accession number(s) can be found in the article/Supplementary Material.
