# Supplementary material for: GlycoIP: an integrated platform for simultaneous and site-specific N/O-glycosylation analysis of human semen
Source: Front Chem. 2025 May 19;13:1569561. doi: 10.3389/fchem.2025.1569561 (PMC12127192; doi:10.3389/fchem.2025.1569561)

**Supplementary Material**

**GlycoIP: An Integrated Platform for Simultaneous and Site-specific N/O-glycosylation Analysis of Human Semen**

Gaoshu Yan^1, #^, Fei Cai^2, #^, Keliang Wu^2^, Qingyuan Cheng^3^, Yong Zhang^2, *^, Fan Lin^1, *^

^1^Department of Radiology, Radiation Oncology Key Laboratory of Sichuan Province, Sichuan Clinical Research Center for Cancer, Sichuan Cancer Hospital & Institute, Sichuan Cancer Center, Affiliated Cancer Hospital of University of Electronic Science and Technology of China, Chengdu 610040, China.

^2^Department of Nephrology and Institutes for Systems Genetics, Frontiers Science Center for Disease-Related Molecular Network, West China Hospital, Sichuan University, Chengdu 610041, China.

^3^Department of Andrology and Sichuan Human Sperm Bank, West China Second University Hospital, Sichuan University, Chengdu 610041, China.

^#^G.Y. and F.C. contributed equally to the work.

***Corresponding authors.**

**Yong Zhang - Address:** No. 1, Keyuan 4th Road, Gaopeng Avenue, Hi-tech Zone, Chengdu 610041, China; **E-mail**: [nankai1989@foxmail.com](mailto:nankai1989@foxmail.com)/zhangyong0809@wchscu.edu.cn.

**Lin Fan - Address:** No. 55, Section 4, South Renmin Road, Wuhou District, Chengdu 610040, China; **E-mail:** fanlin@scszlyy.org.cn.

**Table of Contents:**

**Figure S1.** SDS-PAGE analysis of human sperm and seminal plasma proteins.

**Figure S2.** GO analysis of human sperm O-glycoproteins.

**Figure S3.** GO analysis of human seminal plasma O-glycoproteins.

**Table S1.** Human semen examination results from all volunteers.

**Table S2.** Identified intact N-glycopeptides from human semen.

**Table S3.** Identified N-glycosites from human semen.

**Table S4.** Identified intact O-glycopeptides from human semen.

**Table S5.** Site-specific glycosylation analysis of distinctive proteins from human semen.

**Figure S1.** SDS-PAGE analysis of human sperm and seminal plasma proteins.


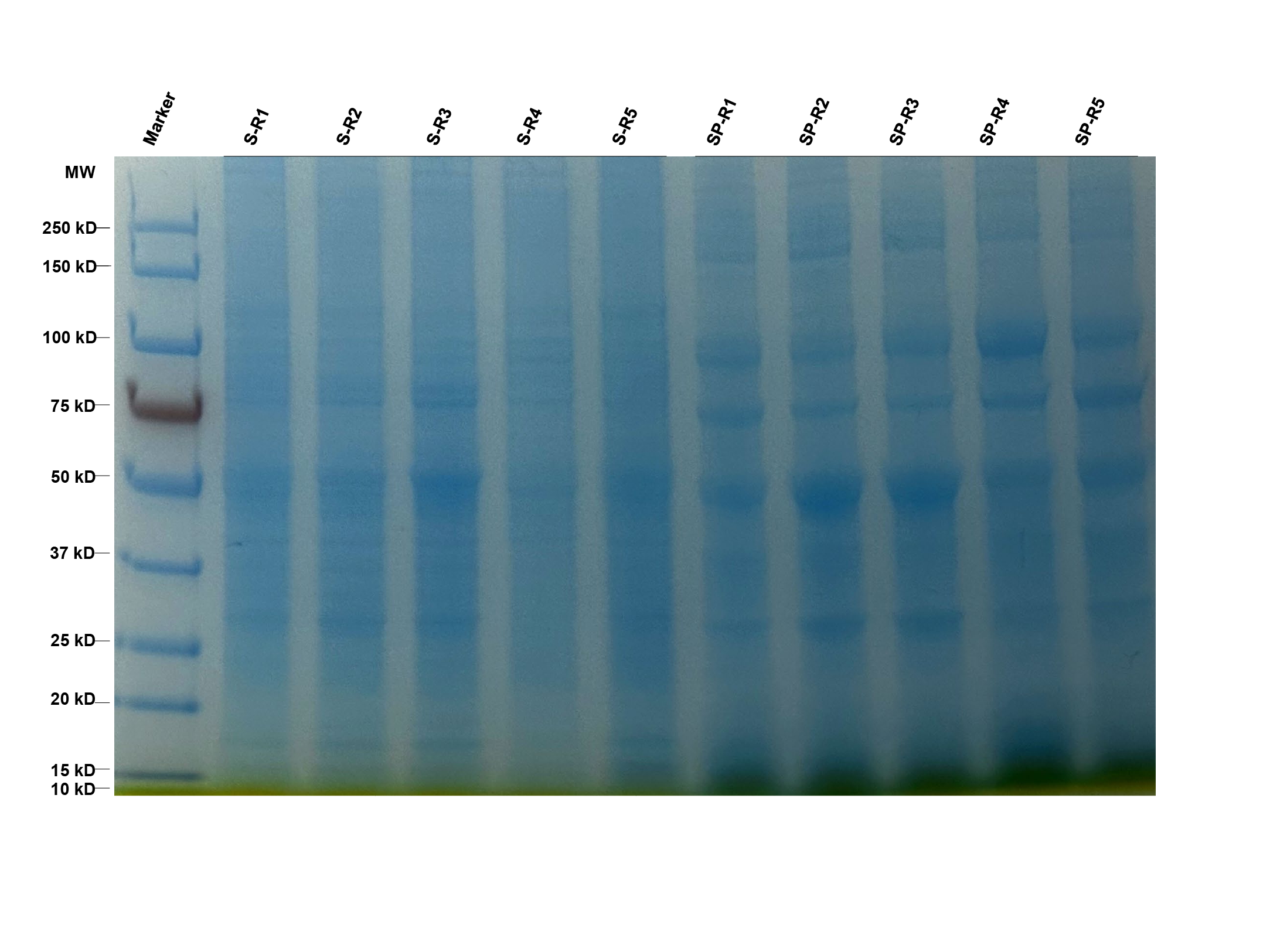


**Figure S2.** GO analysis of human sperm O-glycoproteins.


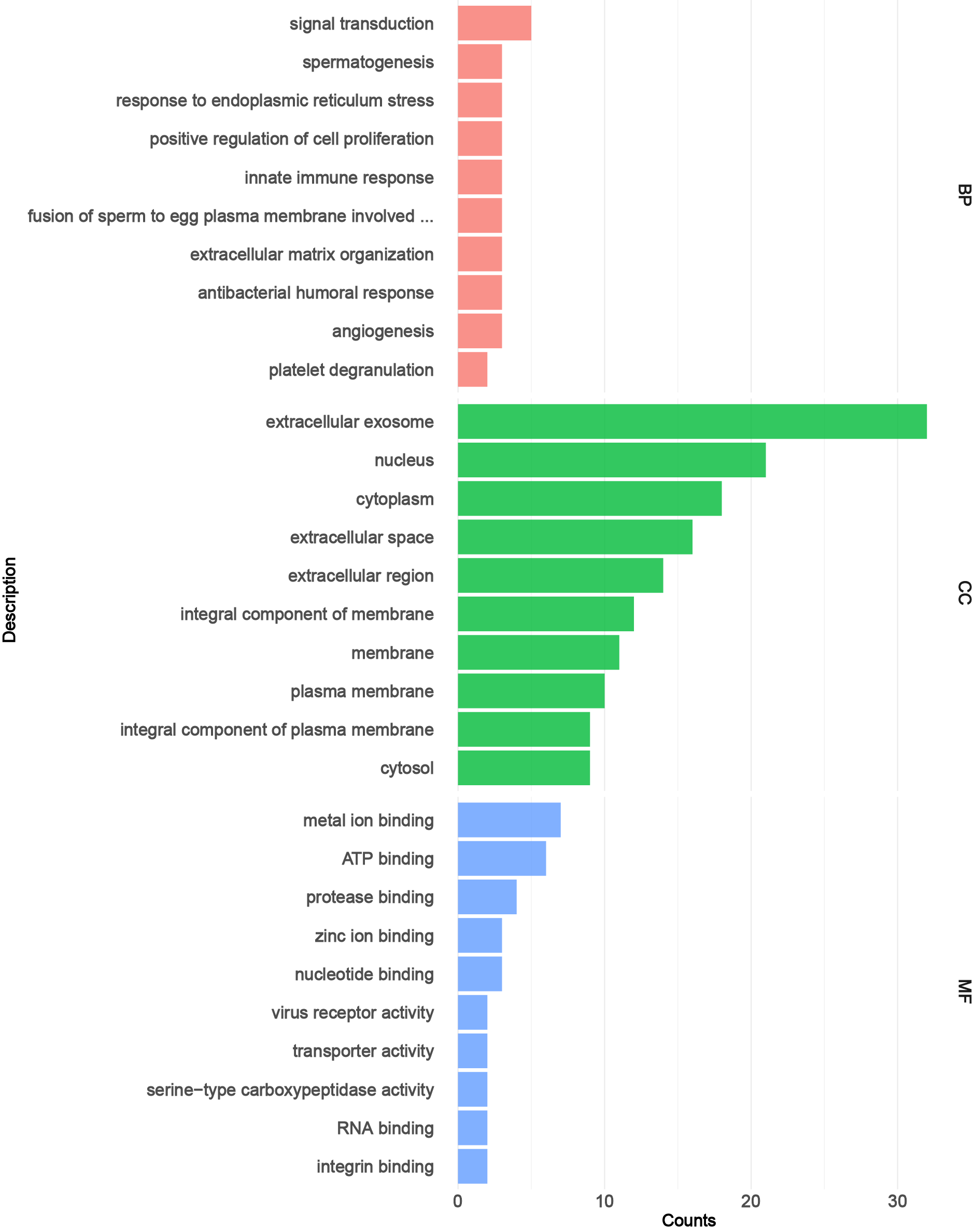


**Figure S3.** GO analysis of human seminal plasma O-glycoproteins.


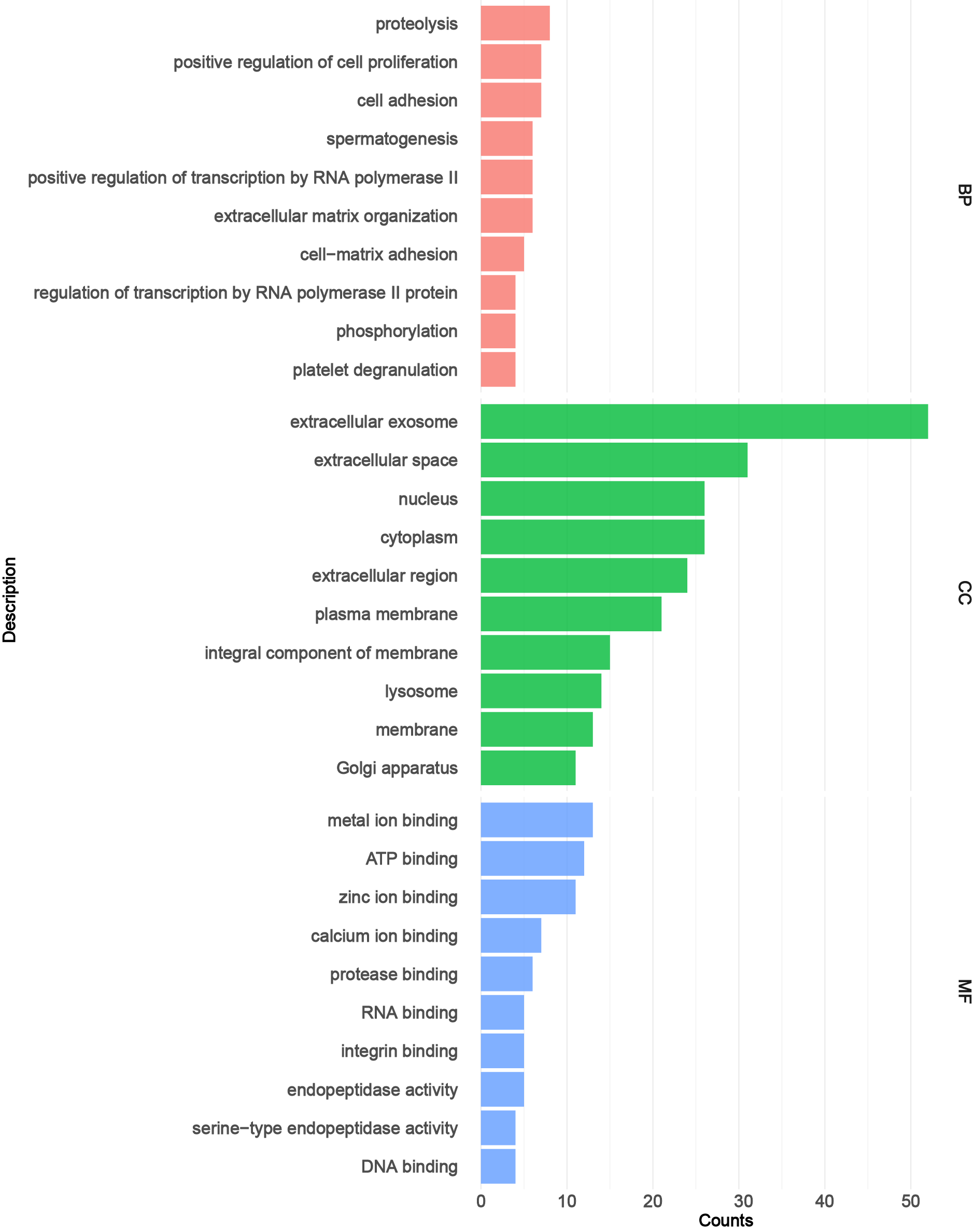

Supplement: Supplementary file 3 [file DataSheet1.doc]
